# Building Global Capacity to Conduct Pathology-Based Postmortem Examination: Establishing a New Training Hub for Minimally Invasive Tissue Sampling

**DOI:** 10.1093/cid/ciab765

**Published:** 2021-12-15

**Authors:** Christina R Paganelli, Lindsay Parlberg, Norman J Goco, Jana M Ritter, Roosecelis B Martines, Sherif R Zaki, Edwin Walong, Washington Ochieng, Dennis Inyangala, Walter Barake, Cyrus Wachiury, Natalia Rakislova, Lorena Marimon, Melania Ferrando, Jaume Ordi, Elizabeth McClure

**Affiliations:** 1 Research Triangle Institute (RTI) International, Seattle, Washington, USA; 2 RTI International, Research Triangle Park, North Carolina, USA; 3 Division of High-Consequence Pathogens and Pathology, Centers for Disease Control and Prevention, Atlanta, Georgia, USA; 4 Anatomic Pathology Unit, Department of Human Pathology, School of Medicine, University of Nairobi, Nairobi, Kenya; 5 Farewell Home Department, Kenyatta National Hospital, Kenya Laboratory Medicine Department, Nairobi, Kenya; 6 ISGlobal, Barcelona Institute for Global Health, Hospital Clínic-Universitat de Barcelona, Barcelona, Spain

**Keywords:** mortality surveillance, capacity building, low- and middle-income countries, minimally invasive tissue sampling, training

## Abstract

**Background:**

Minimally invasive tissue sampling (MITS), an alternative to complete diagnostic autopsy, is a pathology-based postmortem examination that has been validated in low- and middle-income countries (LMICs) and can provide accurate cause of death information when used with other data. The MITS Surveillance Alliance was established in 2017 with the goal to expand MITS globally by increasing training capacity, accessibility, and availability in LMICs. Between January 2019 and May 2020, the MITS Surveillance Alliance convened a multidisciplinary team of technical advisors to attain this goal.

**Methods:**

This article describes the process used to develop criteria and identify an optimal location for a MITS training hub, establish a cadre of LMIC-based trainers, refine standardized MITS sample collection protocols, develop a training program, and release a telepathology platform for quality assessment of MITS histological samples.

**Results:**

Results include the creation of a training hub and curriculum, with a total of 9 pathologists and technicians trained as part of the training of the trainers. Those trainers trained 15 participants from seven MITS projects representing 6 LMICs trained in MITS sample collection. The 15 participants have gone on to train more than 50 project-level staff in MITS sample collection.

**Conclusions:**

Lessons learned include an appreciation for using an iterative process for establishing standardized procedures, creating opportunities for all stakeholders to deliver critical feedback, and highlighting the importance of complementing in-person trainings with ongoing technical assistance.

Accurate mortality data are needed to prioritize health programs and guide health policies. As a result of limited pathology and laboratory resources and insufficient numbers of adequately trained personnel to conduct postmortem investigations in low- and middle-income countries (LMICs), there is inadequate use of the complete diagnostic autopsy (CDA), the gold standard for postmortem assessment of cause of death [1–3]. In low-resource settings when a CDA is not performed the cause of death is often determined based on nonspecific clinical symptoms as reported by family members or medical staff. Without pathologic confirmation, this can lead to the cause of death being attributed inaccurately. Minimally invasive tissue sampling (MITS), a pathology-based postmortem examination technique, which uses biopsy needles for sampling key organs and body fluids, can often be performed with fewer resources than CDA and can be successfully performed by medical technicians and mortuary staff with specialized training [4]. Studies have demonstrated the acceptability and feasibility of MITS in low-resource settings and have validated MITS against the CDA showing that MITS provides accurate data on identifying causes of death and can be used as a proxy for CDA [5].

To support the expansion of pathology-based mortality surveillance, in 2017 the MITS Surveillance Alliance was established as a global multidisciplinary consortium of researchers to expand the use of MITS. The Alliance catalyzed the expansion of MITS by initially funding 12 grants to implement small-scale postmortem studies using MITS, all in LMICs. Most grant recipients lacked previous experience performing MITS sample collection and thus required training.

To date, training on the MITS sample collection technique has been limited and tailored to each study’s specific objectives. Researchers from the Barcelona Institute for Global Health (ISGlobal) as part of the Cause of Death using Minimally Invasive Autopsies (CaDMIA/CaDMIA Plus) projects, organized a training program for the Child Health and Mortality Prevention Surveillance (CHAMPS) Network [4]. The training facility was located within the Maputo Central Hospital, in Mozambique, coordinated by ISGlobal, and had previously trained staff in MITS projects. The ISGlobal research group developed and published operating procedures and protocols to guide training for MITS sample collection [6]. This model was effective but also had limitations that impeded scale-up including the expense of conducting part of the training in high-income countries with a limited number of qualified trainers.

The MITS Surveillance Alliance built on this prior research to establish a new training hub with the goal to expand MITS training capacity and increase accessibility and availability, specifically in LMICs. It is important to acknowledge that although implementing MITS in new contexts requires expertise across a variety of domains including pathology, histology, microbiology, social and behavioral sciences, and cause of death assignment, the primary objective of the training hub was to build capacity in MITS sample collection. MITS sample collection primarily involves the practice and acquisition of new skills; for this reason, training with ample opportunity for skills practice and expert feedback was prioritized. Fundamental to this goal was creating a standardized training program for adult learners that maximized skills practice and acquisition and prepared trainees to cascade the training to additional team members within their awarded projects.

This article describes the MITS Surveillance Alliance strategic approach to expanding MITS sample collection training, including identifying a new MITS training hub, establishing a cadre of LMIC-based MITS trainers, improving standardized collection procedures, developing a detailed training curriculum, and providing ongoing technical assistance to trainees following in-person trainings (Figure 1).

**Figure 1.**
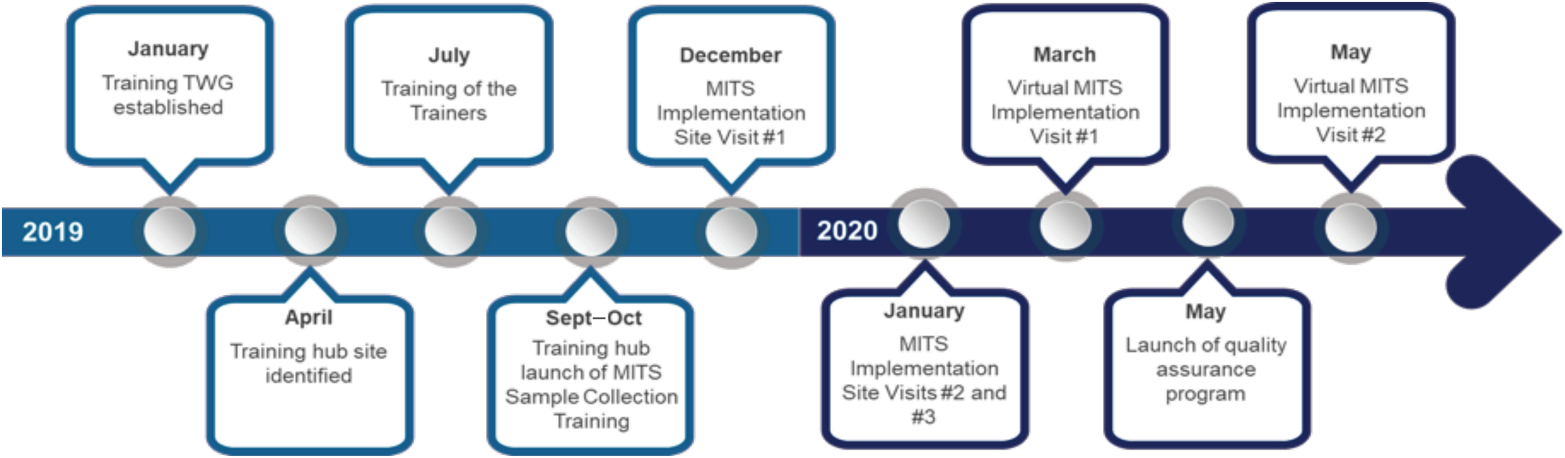
Timeline of MITS training hub. Abbreviations: MITS, minimally invasive tissue sampling; TWG, technical working group.

## METHODS

### Identifying a Training Hub Site

The MITS Surveillance Alliance Training technical working group (TWG) was convened in January 2019 for the purpose of developing a plan to expand the use of MITS globally. The TWG consisted of a diverse group of technical advisors from ISGlobal, Research Triangle Institute (RTI) International, and the US Centers for Disease Control and Prevention’s (CDC’s) Infectious Disease Pathology Branch (Table 1). To identify the optimal location for a training hub the TWG defined criteria for selecting a site including several well-ventilated and lit autopsy rooms, skilled and sufficient staff, an adequate autopsy case load, and ease of access for participants from other LMICs (see Supplementary Material). Following an initial review of potential locations and a site visit where members of the TWG toured facilities and met with institutional leadership, Kenyatta National Hospital (KNH) and the University of Nairobi School of Medicine were selected for the training hub. KNH possessed the necessary facilities and would be relatively easy to access from other LMICs. In addition, the KNH team had recently concluded a study involving MITS and, as a result, employed pathologists and mortuary staff already experienced in MITS sample collection and additional staff sensitized to MITS [7].

**Table 1.** Affiliations and Roles

	Affiliations	Participants	Roles
Training TWG	• CDC, Infectious Disease Pathology Branch • ISGlobal • RTI International	11	• Identify training hub site • Develop and implement Training of Trainers • Refine standard procedures for MITS sample collection • Participate in MITS implementation site visits
Trainers	• Kenyatta National Hospital • University of Nairobi School of Medicine, Department of Pathology	9	• Provide infrastructure, expertise, and commitment to establishing training hub • Provide input into development of MITS sample collection training • Implement MITS sample collection training • Participate in MITS implementation site visits
Trainees	• MITS Alliance grant recipients • Project staff from MITS Alliance member projects	15	• Participate in MITS sample collection training • Train additional project staff in MITS sample collection

Abbreviations: CDC, Centers for Disease Control and Prevention; MITS, minimally invasive tissue sampling; RTI, Research Triangle Institute; TWG, technical working group.

### Conducting the Training of the Trainers

Following the selection of the training hub location, a training of the trainers (TOT) curriculum was developed based on a traditional TOT training model in which members of the TWG conducted a training for pathologists, pathologists in training, and pathology technicians, establishing a regionally based cadre of MITS trainers. Learning objectives for the TOT were developed and agreed upon by the TWG. The objectives were then mapped to the MITS Sample Collection training curriculum implemented by ISGlobal for prior MITS projects. In addition to content directly related to collecting MITS samples, objectives specifically targeting best practices in training professional adults were identified; sessions addressing the principals of adult learning, skills for effective facilitation, and methods for supporting multiprofessional learning were integrated into the TOT curriculum (Table 2) [8].

**Table 2.** MITS Sample Collection Training of the Trainers Curriculum Outline

□ Introduction to MITS in Global Health □ Review of important MITS studies □ Orientation to MITS kits contents and the MITS Sample Collection Standard Operating Procedure □ Biosafety in MITS □ The role of anthropometry and external examination □ MITS Sample Collection demonstration and practice □ External examination and sampling of the placenta □ Handling and initial processing of MITS samples after the procedure □ Principles of adult learning and multiprofessional education

Abbreviation: MITS, minimally invasive tissue sampling.

Members of the TWG conducted a 7-day TOT for 3 pathologists and 5 pathology technicians onsite at KNH in July 2019. Two of the 3 pathologists participating in the TOT had previous experience with MITS. The first 2 days were focused on didactic content and adult learning principles. The subsequent 5 days focused on MITS sample collection. Each day started with a skills practice session followed by a short didactic lesson and concluded with a debrief.

Central to establishing a standardized training program was the adoption and refinement of a standard operation procedure (SOP) for MITS sample collection. Using aspects of SOPs for MITS sample collection already created for other studies, the TWG developed a draft SOP that was piloted over the course of the TOT [6, 9]. The SOP sought to address all variations in the MITS sample collection procedure based on differences in age, and the TWG determined that all MITS sample collection trainings would include the entire SOP, regardless of the target populations to be studied by trainees. Over the course of the TOT, the SOP was modified based on observations by TWG members and feedback from the trainers. During the skills practice sessions, a photographer documented individual steps in the sample collection procedure, and those photos were used to complement written instructions in the SOP.

The daily debriefing sessions consisted of collecting structured and unstructured feedback from both TWG members and trainers about the training process and curriculum organization. In addition, a comprehensive summary feedback session to review all final suggested changes to the curriculum and SOP was held on the last day of the TOT. Outcomes of the feedback sessions included converting some of the in-person content to online asynchronous e-learning modules and increasing the total amount of training time dedicated to skills practice. The feedback sessions also led to the development of the customized MITS Specimen Collection form (see Supplementary Material) and the Placental Examination and Collection form (see Supplementary Material). These forms facilitated standardization and documentation of the sampling process. Because future trainees would be expected to train other members of their project in MITS sample collection, the final skills practice session was modified to simulate a training session where trainees would be given an opportunity to act as trainers and practice teaching MITS sample collection.

### Launch of the Training Hub

**Figure 2.**
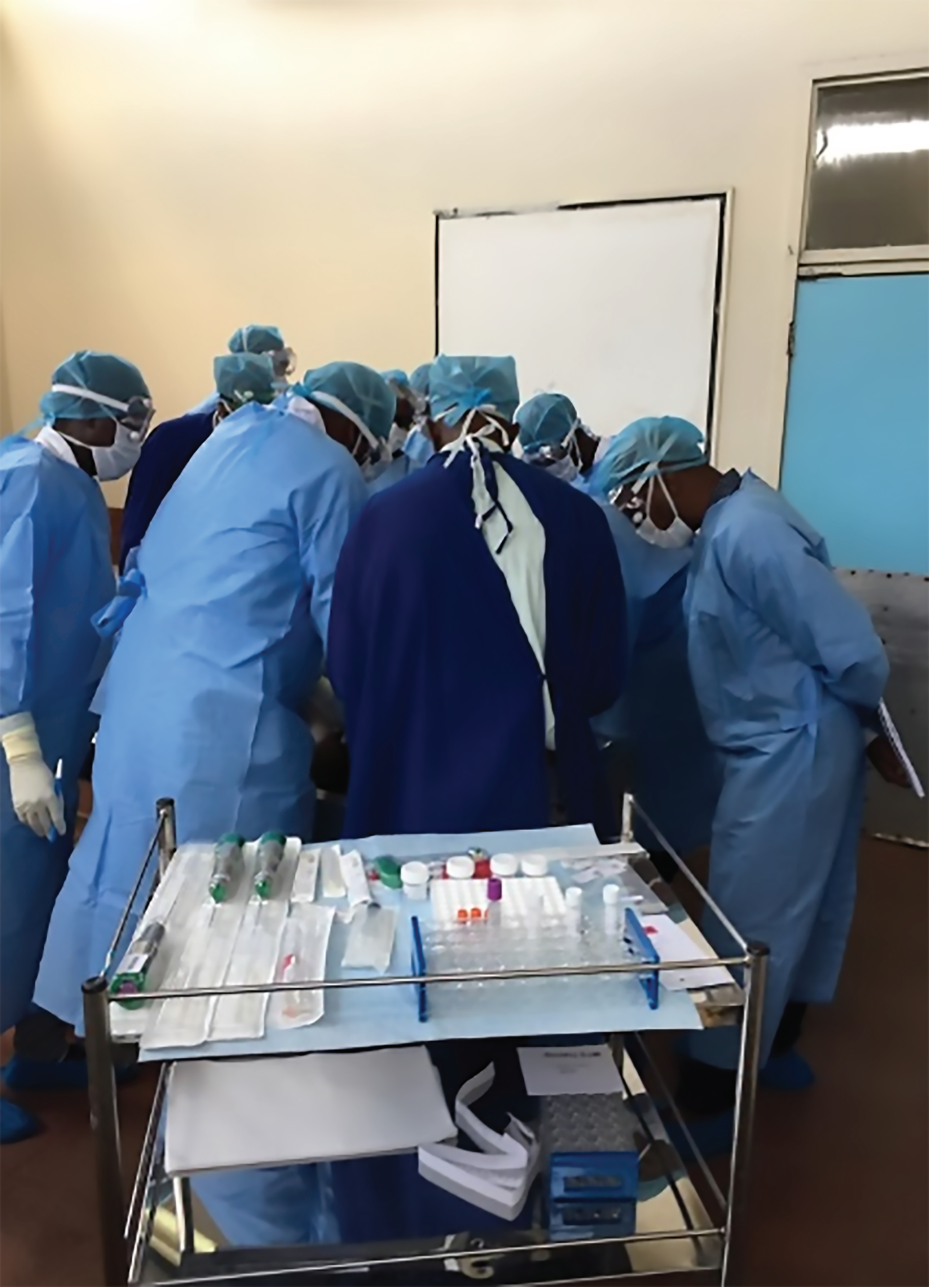
MITS sample collection training, Nairobi, Kenya, September 2019. Abbreviation: MITS, minimally invasive tissue sampling.

In September 2019, trainers trained as part of the TOT began conducting MITS sample collection training for MITS Alliance grant awardees (Figure 2). Trainees consisted of 2 individuals per awarded grant project, including 1 pathologist. A total of 15 representatives from 7 grant projects participated in 1 of 3 separate trainings conducted at KNH over a 2-month period. Prior to attending the 5-day in-person training, trainees were asked to preview the asynchronous online self-study training modules adapted as part of the TOT and review the MITS Sample Collection SOP. Each in-person training consisted of no more than 6 trainees and included a minimum of 2 trainers.

TWG members attended the first and second trainings to provide supportive supervision to the trainers. The supportive supervision included joint daily debriefings to share observations from the day, support to the trainers in preparations for the subsequent day of training, and documentation of observations about the curriculum and training materials. This iterative approach allowed trainers to revise and fine-tune the curriculum over the course of the initial trainings. In addition to TWG members, a pathologist with extensive expertise in MITS training from the CaDMIA/CaDMIA Plus projects in Mozambique was invited to attend a training. This external expertise and feedback offered trainers a different perspective and established a strong foundation for future South-South cooperation.

All trainees completed an evaluation on the final day of the training program (see Supplementary Material). This evaluation prompted trainees to share critical feedback on the program, such as whether the course met the trainee’s needs, and sought feedback regarding the training structure, facilitation, and organization. The evaluation also included space for participants to identify learning needs that were not met as part of the course. Overall trainees strongly agreed that the course objectives were met and the training was thorough, clear, effective, and well organized. Specific feedback noted that the trainer to trainee ratio was adequate, there was ample time for skills practice, and the trainees appreciated the trainers’ positive and supportive training style. Trainees also noted that they would have liked a longer training and that they would benefit from additional opportunities to practice MITS sample collection on the specific population ages that they would be studying for their project.

### Post-training Technical Assistance

Following the conclusion of the in-person trainings, the TWG and trainers collaborated to provide additional technical assistance intended to complement the in-person training. Activities included conducting implementation site visits and establishing a telepathology platform to enable remote quality assessment of MITS sample collection.

### MITS Implementation Site Visits

The site visits were designed to reinforce training and support trainees in MITS implementation and to further strengthen the capacity of trainers following the in-person training. Sites were identified based on complexity of the study, whether the site had previous experience conducting MITS, observations of skill acquisition by the trainers over the course of the in-person training, and the level of existing resources available at the site. Assessment criteria for the site visits were prepared and included prerequisites for the visit, a description of the goals and objectives of the site visit, and a proposed agenda (see Supplementary Material). Using a mentor model site visits were conducted jointly by both the TWG members and trainers. This approach provided opportunities to strengthen the capacity of the trainers to apply their technical expertise in support of trainees and assume increasing levels of leadership over the course of the visits.

Site visits were initially planned for 5 projects. Ultimately, 3 of the 5 were carried out in person, but the remaining 2 were conducted virtually because of the severe acute respiratory syndrome coronavirus 2 (SARS-CoV-2). The first site visit was conducted by members of the TWG over 4 working days in December 2019. Each site visit focused on observations and feedback of MITS sample collection procedures and techniques and the review of already processed histological MITS samples. The TWG observed trainees’ MITS sample collection and provided real-time feedback with suggestions for improving technique and accuracy. The review of processed histological MITS samples as part of the implementation visit emphasized the value in providing feedback and troubleshooting challenges in MITS sample processing. Trainees were encouraged to incorporate suggestions made by the TWG over the course of the visit, and as a result the TWG witnessed immediate improvements at the sites.

After the SARS-CoV-2 pandemic was declared, travel was not feasible, and the remaining 2 site visits were conducted via videoconference. In preparation for these “virtual” visits, prior to the meeting date trainees were asked to create and share videos and photos of their facilities including a detailed mapping of the expected workflow and sample storage. The materials provided by the sites were reviewed by the TWG members in advance of the scheduled videoconference. During the live videoconference, TWG members provided feedback to the site and helped troubleshoot and identify solutions for potential issues related to implementing MITS sample collection locally.

### MITS Sample Quality Assurance Platform

TWG members collaborated in the design and development of an online telepathology platform aimed at supporting trainees in ongoing quality improvement of MITS sample collection and processing for histopathology. The telepathology system was largely based on CDC’s Infectious Diseases Pathology Branch telepathology approach for supporting CHAMPS and other MITS studies [4, 10]. Trainees uploaded images of each tissue type from their initial cases and completed a self-evaluation.

Following the self-evaluation, CDC pathologists used the same criteria to evaluate and document overall case and image-specific feedback. After a case was evaluated, trainees were able to view evaluator feedback and comments.

During the site visits, trainees were oriented to the telepathology system and practiced uploading images and completing the self-evaluation with support from the TWG. Discussion during the site visit subsequently informed the creation of a User Guide and Frequently Asked Questions document.

### Additional Technical Assistance

The MITS Surveillance Alliance convenes quarterly meetings to provide ongoing support to trainees. These meetings have helped sites troubleshoot MITS related challenges, address specific topics such as how SARS-CoV-2 is impacting their ability to conduct MITS, and serve as a forum for sharing experiences and learning. Trainees also participate in monthly virtual domain-specific TWG meetings which create unique opportunities for trainees to interface with peers and more experienced MITS researchers. All trainees have access to a private chat group to connect and interact in real time.

## CONCLUSION

This article describes the approaches used to increase the global availability and accessibility of MITS training by successfully establishing a training hub in an LMIC. We describe in detail the activities carried out to identify a training hub site, conduct a TOT to establish a cadre of MITS trainers, implement MITS sample collection training, and provide post-training ongoing technical support to trainees. There were several valuable lessons learned as part of establishing this training hub.

One lesson was the importance of engaging an experienced and multidisciplinary group of experts with a commitment to expanding the use of MITS at the outset. Prior to the establishment of the training hub, there had been little published on MITS training, few models for implementing MITS training, and only a handful of individuals capable of building new capacity in MITS sample collection. By establishing a Training TWG consisting of individuals from different institutions and disciplines, we were able to capitalize on the collective experience and knowledge regarding MITS sample collection training to establish a well-prepared training hub, refine the standardized sample collection procedure, and launch a comprehensive training curriculum.

A second lesson learned was that there is value in continued communication and support for the trainers following the TOT. This allowed the TWG to identify and promptly address any gaps in knowledge or resources needed to implement MITS sample collection trainings. By observing the KNH team while conducting the first MITS sample collection training, the TWG facilitated the trainers in identifying both successes and areas for improvement. Frequent communication between the TWG and trainers also supported iterative development and revision of the MITS sample collection training curriculum. This approach empowered the trainers to adapt the curriculum based on their observations and assume increasing ownership over trainings.

Similarly, providing ongoing opportunities for technical assistance in MITS implementation to trainees was important and reinforced the skills learned as part of the in-person training. Site visits helped trainees refine and perfect their MITS sample collection technique and improve the quality of sample processing. The telepathology platform established a durable channel for trainees to receive expert feedback on the quality of MITS sample collection and processing.

And lastly, by creating enduring training materials including the didactic lessons adapted to self-paced e-learning modules and a well-illustrated SOP, the initial impact of training was extended to support trainees to expand MITS sample collection capacity within their sites and train additional members of their project team.

### The Future of MITS Training

Until March 2020, plans for further utilizing the MITS training hub included conducting the remaining site visits for trainees and supporting additional trainings. However, the global SARS-CoV-2 pandemic has significantly curtailed planned activities. Curriculum modifications to support remote learning have been made and include utilizing new methodologies. A complete series of comprehensive step-by-step training videos teaching MITS sample collection was created by the trainers. The videos and didactic content are discussed in depth via a series of interactive videoconferences. Trainees use mobile devices and hotspots to demonstrate and record their MITS sample collection technique. Trainers review each recording and share feedback, troubleshoot challenges, and debrief with trainees in advance of the next skills practice over videoconference. The other methods for supporting MITS training, including the TWGs, the telepathology platform and regular meetings with trainees, continue without interruption. The TWG will continue to innovate and adapt to expand MITS training accessibility and availability within existing limitations.

### Global Significance

Accurate mortality data are needed to prioritize health programs and interventions to guide health policies. The SARS-CoV-2 pandemic highlights the urgent need for pathology-based cause of death determination. MITS is comparable to CDA and can be implemented in LMICs with lower costs and fewer resources. By establishing a training hub and cadre of trainers for MITS sample collection, MITS training capacity and accessibility has been expanded, making pathology-based postmortem examination more attainable, especially within LMICs.

## Supplementary Data

Supplementary materials are available at *Clinical Infectious Diseases* online. Consisting of data provided by the authors to benefit the reader, the posted materials are not copyedited and are the sole responsibility of the authors, so questions or comments should be addressed to the corresponding author.

ciab765_suppl_Supplementary_Materials

## Notes

***Acknowledgments.*** The authors acknowledge the University of Nairobi School of Medicine, and the Kenyatta National Hospital Administrative Staff for their support launching of the MITS Alliance Training Hub.

***Authors’ contributions.*** C. P. led the development and writing of the manuscript. L. P. provided significant input on manuscript structure, outline, and content. All authors reviewed the manuscript and provided critical feedback which has been incorporated into the submission.

The authors acknowledge this work has been previously presented as an ePoster at the American Society of Tropical Medicine and Hygiene 2020 Annual Meeting.

***Supplement sponsorship.*** This supplement is sponsored by MITS Surveillance Alliance Secretariat, led by RTI International, with funding from the Bill and Melinda Gates Foundation.

***Financial support.*** This work was supported by the MITS Surveillance Alliance. This work was supported, in whole or in part, by the Bill & Melinda Gates Foundation (grant number OPP1180554). Under the grant conditions of the Foundation, a Creative Commons Attribution 4.0 Generic License has already been assigned to the Author Accepted Manuscript version that might arise from this submission.

***Potential conflicts of interest.*** The authors: No reported conflicts of interest. All authors have submitted the ICMJE Form for Disclosure of Potential Conflicts of Interest. Conflicts that the editors consider relevant to the content of the manuscript have been disclosed.
