# Supplementary material for: Building Global Capacity to Conduct Pathology-Based Postmortem Examination: Establishing a New Training Hub for Minimally Invasive Tissue Sampling
Source: Clin Infect Dis. 2021 Dec 15;73(Suppl 5):S390–5. doi: 10.1093/cid/ciab765 (PMC8672740; doi:10.1093/cid/ciab765)
Supplement: ciab765_suppl_Supplementary_Materials [file ciab765_suppl_supplementary_materials.docx]

# Supplementary Material: Training HUB Assessment Criteria

- Institutional and team commitment
- Commitment of administration
- Commitment of the training team
- Adequate facilities for theoretical sessions
- Classroom with capacity to host 10 people
- Data show (and computer) for slide presentations
- Adequate facilities for practical sessions
- Clean and equipped autopsy room with capacity to host six to seven people
- Pathology laboratory with room to host one to two technicians and equipped with tissue processor (ideally rotation type), manual staining battery
- Additional microtome to be devoted for training (at least, partially during the training courses)
- Daily access to autopsies
- Minimum of one to two per day; ideally representing different age populations (stillbirth/neonates, pediatric and adults)
- Medical and technical staff with availability to conduct training courses of 1- to 2-week duration. The required profiles include:
- Pathologists
- Pathology technicians
- Personnel to serve as liaison for coordinating and planning training courses
- Administrative/logistical support

# Supplementary Material: MITS Specimen Collection Form


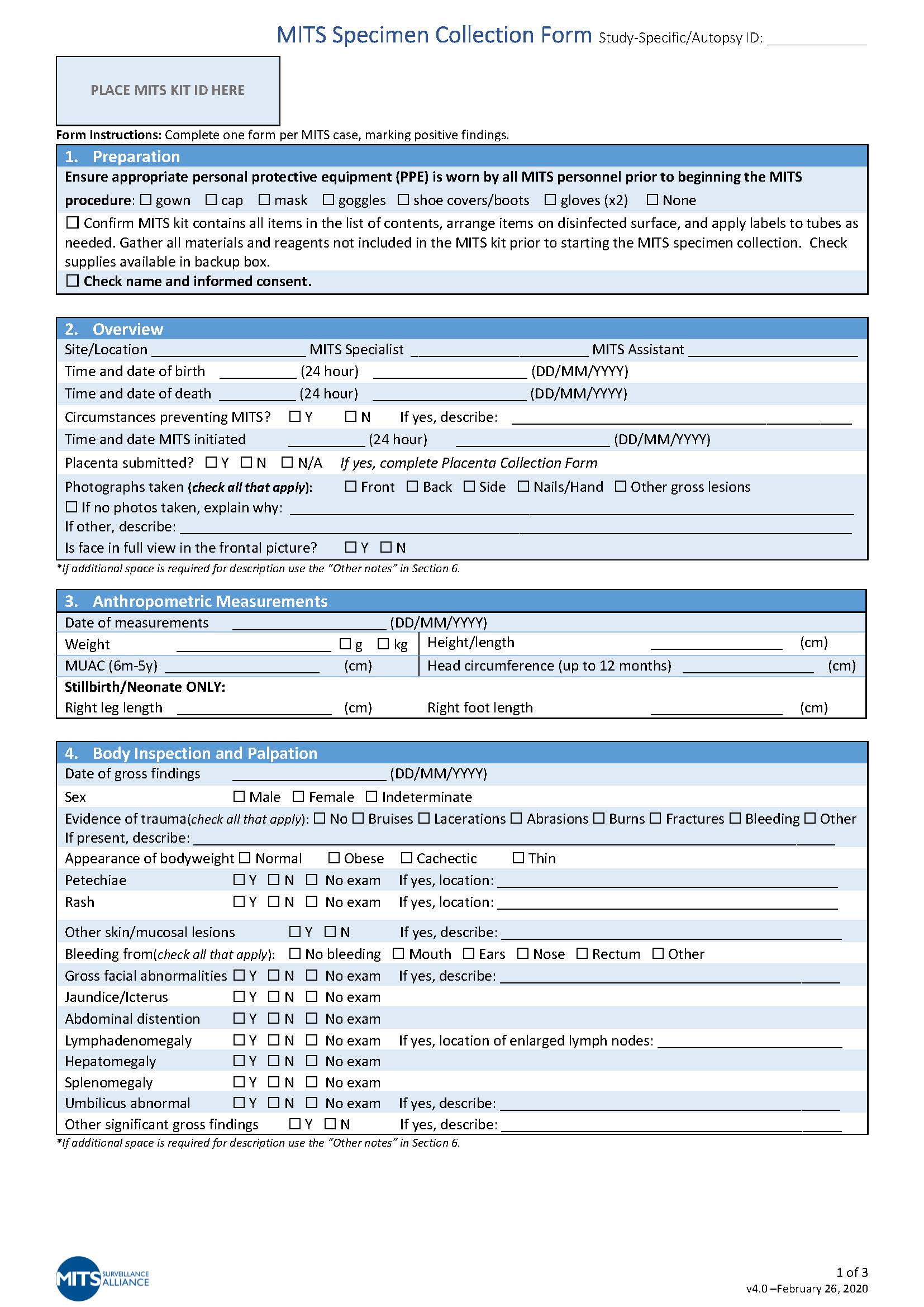


**
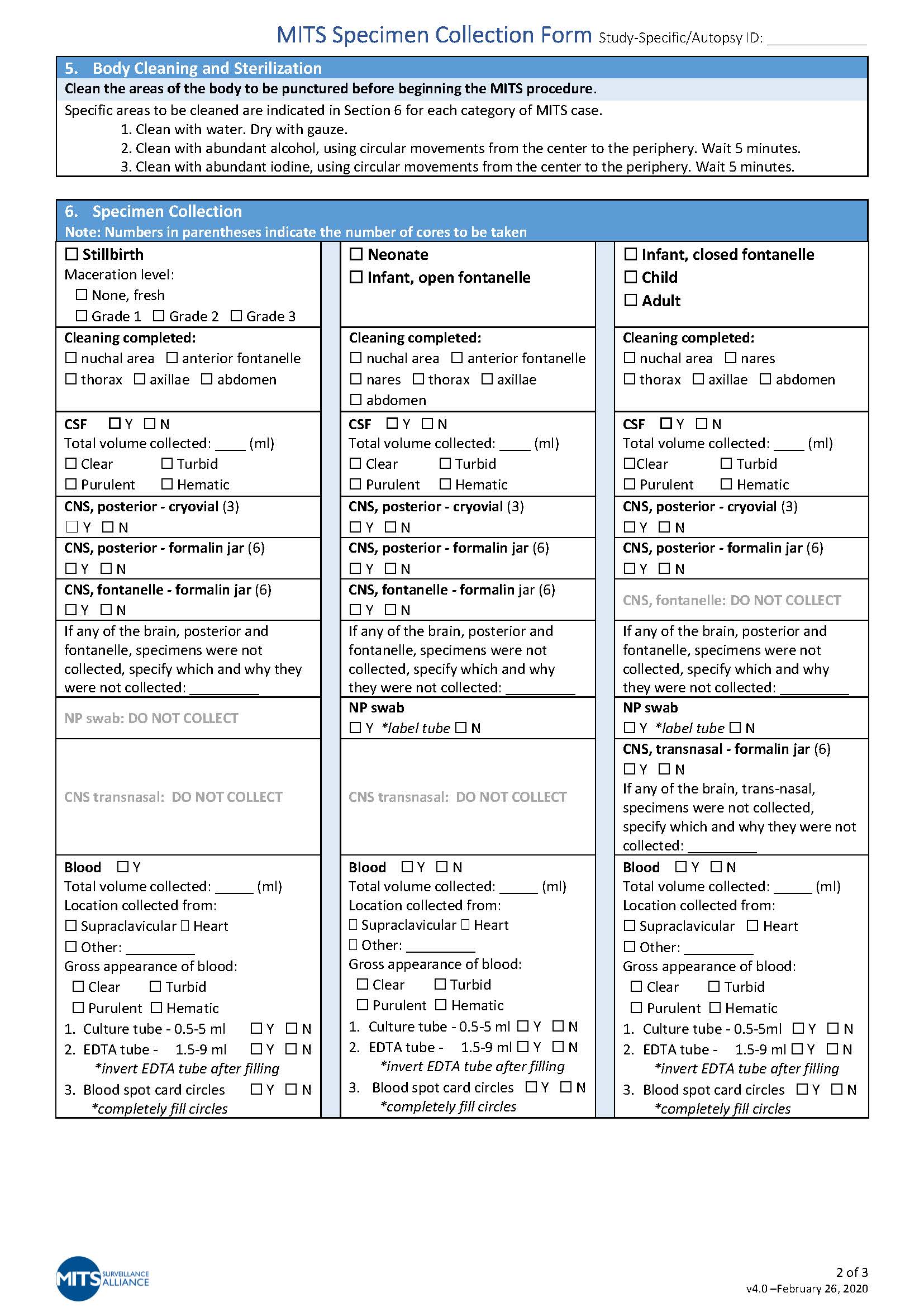
**

**
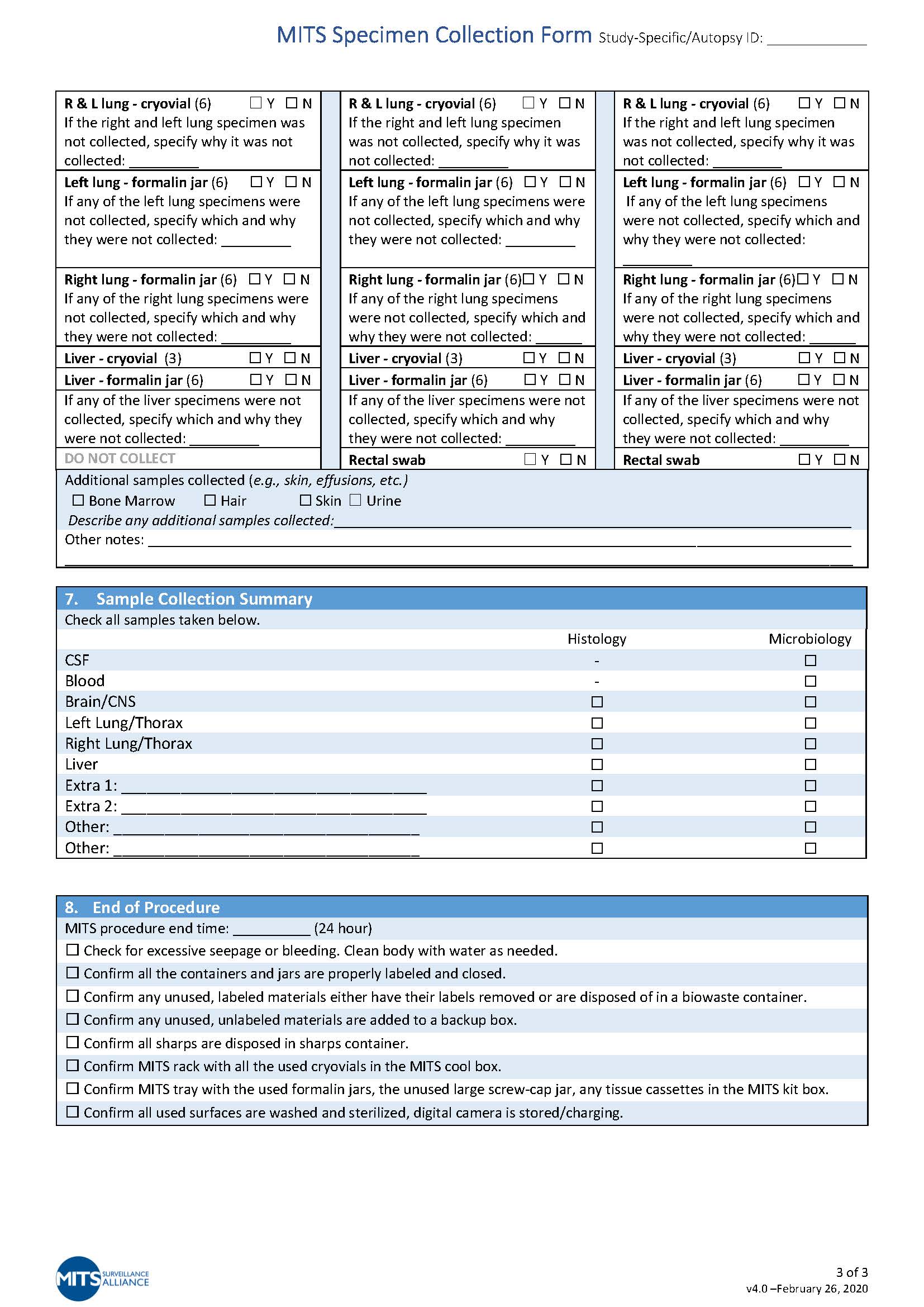
**

# Supplementary Material MITS Placenta Collection Form


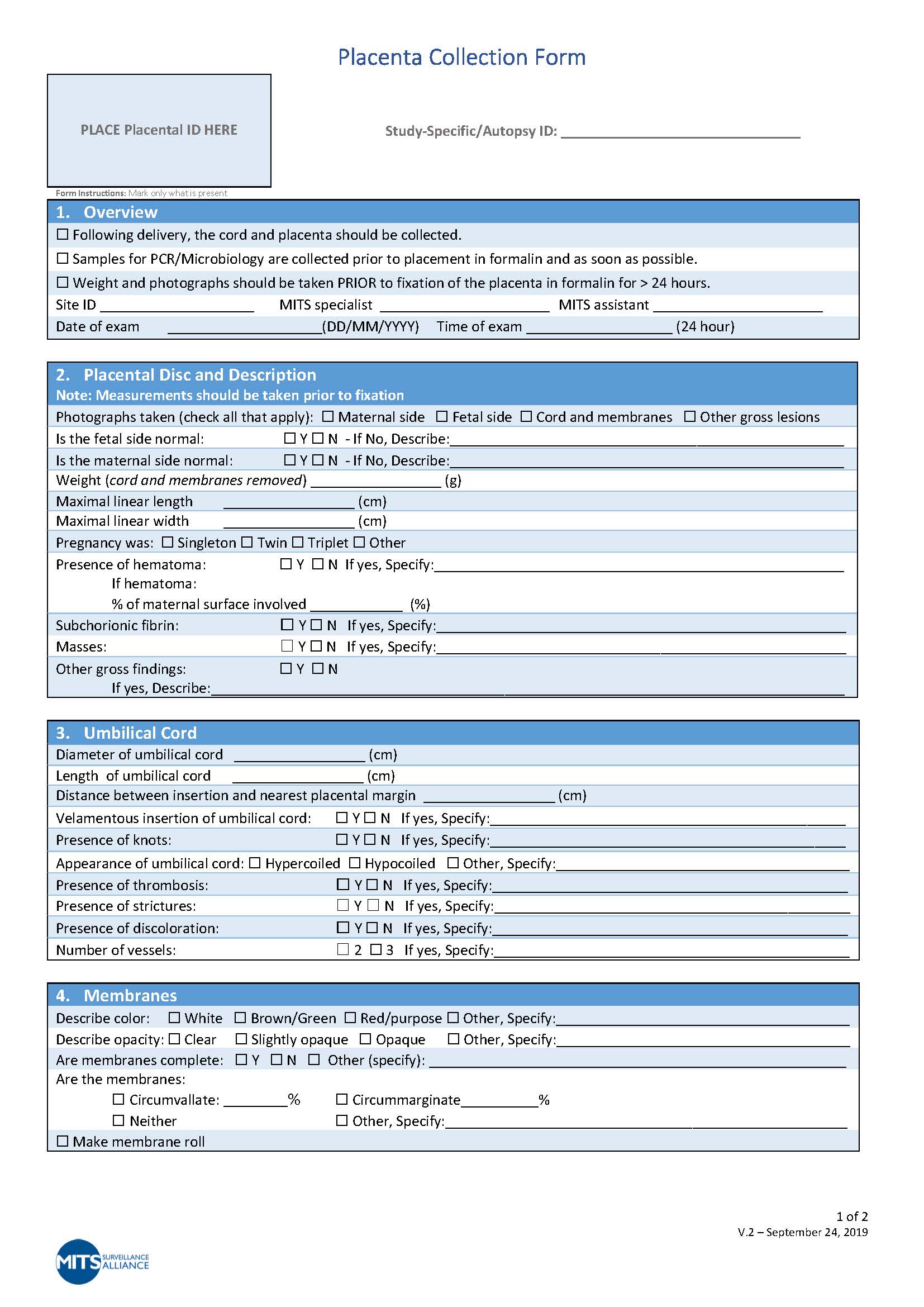


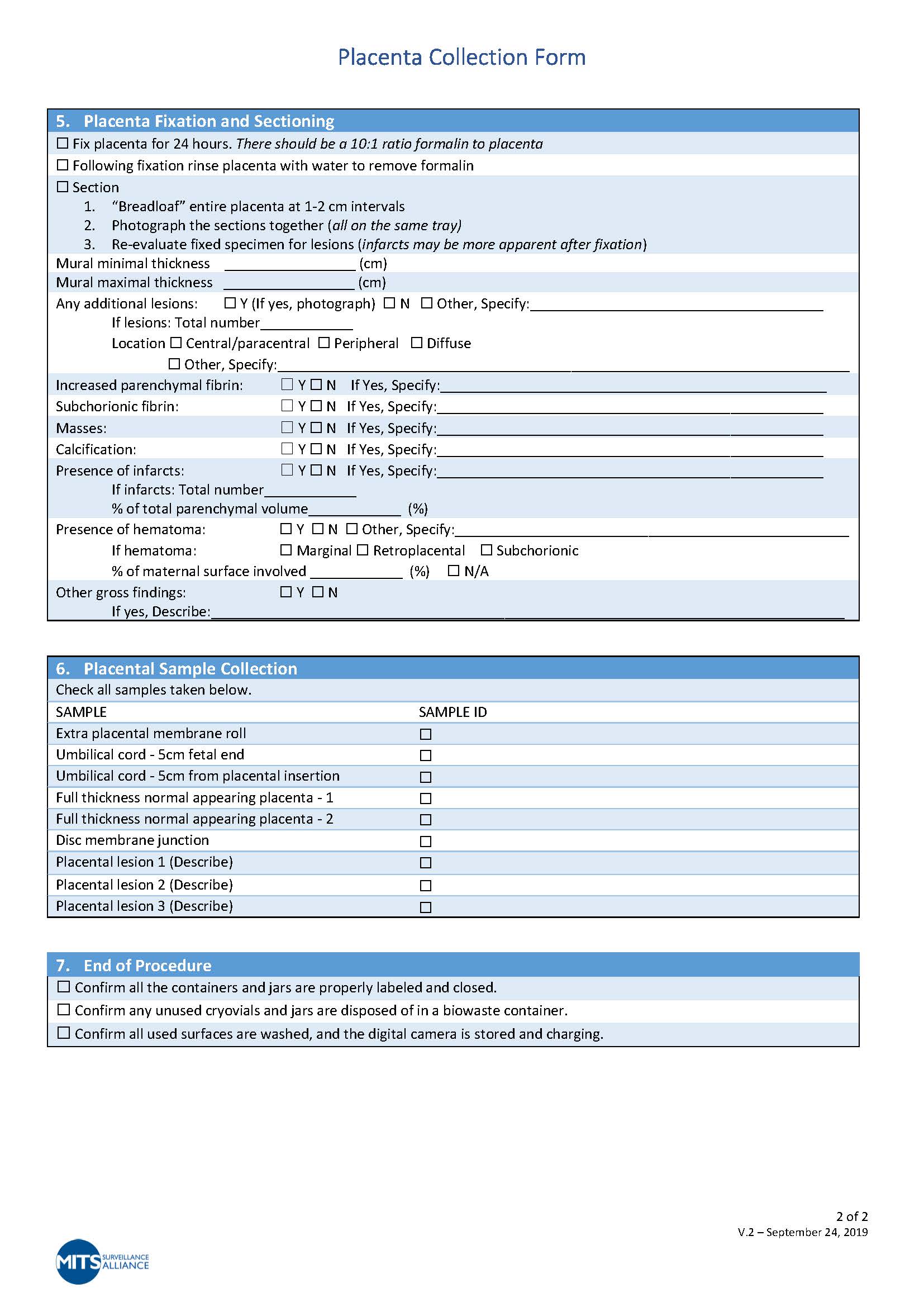


# Supplementary Material: Sample Collection Training Evaluation


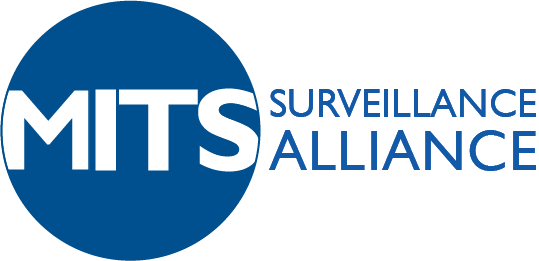


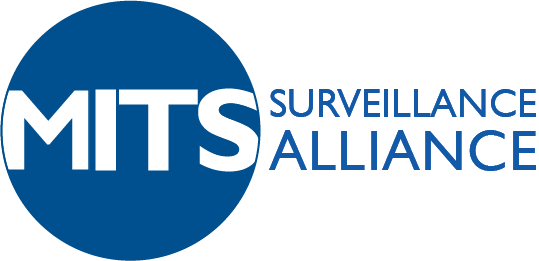


MITS Training Evaluation Form

Training Dates:_______________________

**Workshop Evaluation**

## A. Course design (Circle the number to indicate your level of agreement/disagreement with each of the aspects of course design.)

Strongly disagree Strongly agree

1. The program content met my needs. 1 2 3 4

2. Length of the course was adequate 1 2 3 4

3. What did you like most about the course?

4. What specific things did you like least about the course?

## B. Course objectives:

Circle the number to indicate your level of agreement/disagreement with the degree to which course objectives were met

|  | Strongly disagree Strongly agree |
| --- | --- |
| Name each component of the MITS kit and state its intended use | 1 2 3 4 |
| Demonstrate correct CSF, CNS, NP, Blood, Lung, Liver, Skin and Rectal MITS sampling technique | 1 2 3 4 |
| State age specific variations in the MITS Alliance SOP | 1 2 3 4 |
| Demonstrate correct placenta sampling technique | 1 2 3 4 |
| Demonstrate and maintain proper biosafety precautions | 1 2 3 4 |
| Create a draft schematic for MITS operations in their own projects | 1 2 3 4 |
| Identify the presence of external malformations and congenital anomalies | 1 2 3 4 |

## C. Evaluation of training structure, facilitation and organization

|  | Strongly disagree Strongly agree |
| --- | --- |
| Content was presented in an organized way | 1 2 3 4 |
| Content was presented clearly and effectively | 1 2 3 4 |
| Was responsive to questions/comments | 1 2 3 4 |
| Teaching aids/audiovisuals were used effectively | 1 2 3 4 |
| Teaching style was effective | 1 2 3 4 |
| Content met stated objectives | 1 2 3 4 |

Overall, I would rate this workshop as:

___Excellent

___Good

___Average

___Poor

Other learning needs: (List any other topics you would be interested in for the future)

# Supplementary Material: MITS Implementation Site Visit Assessment Criteria

## MITS Implementation Site Visit Assessment Criteria

Visit Goal: The goal of MITS implementation visits is to provide technical assistance to incentive grantees in MITS implementation as part of carrying out its proposed research.

Visit Objectives: Technical advisors will review and provide feedback and guidance in the following domains:

1. Study design and objectives

2. Project administration and management

3. Study logistics

4. MITS sample collection (observe technique)

5. MITS sample histology and microbiology processing

6. Process for determining cause of death

7. MITS Alliance QA/telepathology expectations and use

### Implementation Visit Prerequisites:

- Ethics approvals obtained
- Completion of MITS Sample Collection Training by two or more members of the study team
- Study Sample Collection SOP finalized
- Kit development form completed and approved
- First MITS kit shipment received
- Additional study staffed trained
- Initiated recruitment; conducted one or more MITS cases
- Initial samples from MITS cases processed and analyzed for histology and microbiology
- Initial plans for cause of death determination process and participants involved

### Project Team Participants

1. Project PI(s)

2. MITS specialists/assistants (if other than PIs)

3. Social scientist/community sensitization lead

4. Pathologist responsible for evaluating samples

5. Microbiologist responsible for evaluating samples

### Project Teams Should Be Prepared To Present The Following:

1. Description of sensitization activities conducted and any future plans

2. Description of subject recruitment and enrollment (inclusion/exclusion criteria) procedures

3. Description of histology and microbiology SOPs

4. Description of the process for determining cause of death

5. Tissue blocks and slides from initial MITS cases

6. Histopathology and microbiology reports from initial MITS cases

### Other Preparations

- In order to provide MITS sample processing and analysis feedback if teams do not have processed samples from MITS study cases to review, they should be prepared to collect samples from a non-study case (unidentified or abandoned cadaver) and process per their SOP prior to visit start date.

### Implementation Visit Assessment Areas

#### Study design and objectives

a. MITS sample collection SOP supports the study objectives

b. There are sufficient resources (material and human) to analyze samples

i. Storage

ii. Reagents

iii. Lab staff

c. Analysis plan meets study objectives

#### Project administration and management

a. Project management system that tracks milestones and payments

b. Adequate data management system

i. Secure, comprehensive, backed-up

c. Community sensitization activities

i. Facility medical staff

ii. Family members, next of kin

iii. Community leaders

#### Study logistics

a. System for managing MITS Kit stock

i. Tracking shipments, arrivals and usage

ii. Adequate and secure storage

b. Location of postmortem room

i. Near a door with vehicular access (if appropriate)

ii. Adequate ventilation and light

iii. Away from clinical, kitchen and dining areas

iv. Restricted from public access

v. Located proximate to hospital

vi. Connected to dirty/disposal room

vii. Clearly marked biohazard

c. Clean activity areas

i. Reception area/waiting room

ii. Staff dressing room (gender specific?)

iii. Linen/materials storage

iv. Specimen storage

d. Transit areas

i. Appropriate (temp, security) storage for body

ii. Biohazard waste disposal

e. Workflow

i. Workflow between rooms minimizes movement from dirty to clean areas

ii. Adequate handwashing and waste disposal receptacles appropriately placed

#### MITS sample collection

a. Preparation of MITS case

i. PPE use

1. Appropriate donning of PPE

ii. Clear identification of roles and responsibilities for sample collection in each case

iii. Appropriate arrangement of kit supplies

iv. Photographs

v. Anthropometric measurements

vi. Body inspection and palpation

vii. Body cleaning and sterilization

b. Sample collection technique

i. Satisfactory sample collection

1. CSF

2. CNS

a. Occipital

b. Fontanelle

c. Trans-nasal

3. Blood

4. Lungs

5. Liver

6. Additional samples

c. Sample labeling and storage

i. Tissue or fluid sample placed in correct container and labeled properly

ii. Microbiological samples stored in cool box

d. End of procedure

i. Disposal of unused materials in biohazard containers

ii. Disposal of sharps in designated sharps containers

iii. Surfaces cleaned with 70% ETOH

iv. PPE removed and discarded in biohazard before exiting room

v. Hands washed outside of MITS room

#### MITS histology and microbiology processing and analysis

a. Pre-analysis phase (processing, embedding, sectioning and staining)

i. Review protocol (processing)

ii. Lab tour

iii. Observe prepared blocks and slides for assessment of embedding and sectioning

iv. Staining-observe sides and/or during QA module

b. Analysis/Internal and external QA for processing quality (not interpretation)

i. Joint slide review

ii. Pathology checklist

c. Sharing results with team

i. Template/model for reporting checklist

ii. Each tissue Dx

iii. Overall summary most significant thing(s) in case

#### Process for determining cause of death

a. Identified participants and process for ascertaining cause of death

#### Quality assurance using telepathology systems

a. Functional microscope and camera/scanner

b. Staff competent in use of microscope/camera/scanner

c. Staff able to log-in, upload and evaluate slide images

d. Staff able to review QA evaluation
